# Pregnancy Outcomes in Non-Criteria Obstetric Antiphospholipid Syndrome: Analysis of a Cohort of 91 Patients

**DOI:** 10.3390/jcm13247862

**Published:** 2024-12-23

**Authors:** Sara Beça, Núria Baños, Maria Borrell, Estíbaliz Ruiz-Ortiz, Albert Pérez-Isidro, Ricard Cervera, Joan Carles Reverter, Dolores Tàssies, Gerard Espinosa

**Affiliations:** 1 Department of Autoimmune Diseases, Reference Centre for Systemic Autoimmune Diseases (UEC/CSUR) of the Catalan and Spanish Health Systems-Member of ERNReCONNET, Hospital Clínic, University of Barcelona, 08036 Barcelona, Catalonia, Spain; sidossantos@clinic.cat (S.B.); rcervera@clinic.cat (R.C.); 2 Institut d’Investigacions Biomèdiques August Pi i Sunyer (IDIBAPS), 08036 Barcelona, Catalonia, Spain; nbanos@clinic.cat (N.B.); esruiz@clinic.cat (E.R.-O.); alperezi@clinic.cat (A.P.-I.); reverter@clinic.cat (J.C.R.); dtassies@clinic.cat (D.T.); 3 Department of Maternal-Fetal Medicine, BCNatal, Barcelona Centre for Maternal-Fetal and Neonatal Medicine, Hospital Sant Joan de Déu and Hospital Clínic, University of Barcelona, 08028 Barcelona, Catalonia, Spain; mborrellb@clinic.cat; 4 Department of Immunology, Centre de Diagnòstic Biomèdic (CDB), Hospital Clínic, University of Barcelona, 08036 Barcelona, Catalonia, Spain; 5 Department of Hemotherapy and Hemostasis, Hospital Clínic, University of Barcelona, 08036 Barcelona, Catalonia, Spain

**Keywords:** non-criteria obstetric antiphospholipid syndrome, adverse pregnancy outcomes, aspirin, heparin

## Abstract

**Background**: The clinical and laboratory features of patients with non-criteria obstetric antiphospholipid syndrome (NC-OAPS), as well as their pregnancy outcomes and ideal treatment are not clearly determined. The aim of this study is to describe the characteristics and outcomes of pregnancies in NC-OAPS and compare them with an obstetric APS (OAPS) cohort. **Methods**: This is a retrospective study conducted on a cohort of women referred to a high-risk obstetric unit of a tertiary hospital. Women that were classified as having OAPS or NC-OAPS were included and compared in terms of clinical and laboratory characteristics, management, and subsequent pregnancy outcomes. **Results**: We identified 107 women with 143 pregnancies, 91 with NC-OAPS and 16 with OAPS. There were no differences in demographic features between both groups. Women with NC-OAPS were more likely to have recurrent implantation failure and were predominantly positive for a single antiphospholipid antibody (aPL) subtype. Both groups were treated similarly (low dose aspirin plus low molecular weight heparin in 87.4% of NC-OAPS and 83.3% of OAPS, *p* > 0.05). Live birth rate (82.4% and 75.0%, respectively, *p* > 0.05) and adverse pregnancy outcomes (31.6% vs. 37.5%, *p* > 0.05) in subsequent pregnancies during follow-up were also similar between groups. **Conclusions**: This study revealed differences in the previous pregnancy morbidity and aPL profiles in women with NC-OAPS and OAPS, although the therapeutic approach and the outcomes of subsequent pregnancies were similar in both groups.

## 1. Introduction

Antiphospholipid syndrome (APS) is characterized by the presence of antiphospholipid antibodies (aPLs) and clinical manifestations, such as vascular thrombosis and pregnancy morbidity. For research purposes, APS classification criteria have been created and updated [1,2,3]. Currently, the most widely used are the Sydney criteria [2]. In daily clinical practice, particularly in the case of obstetric APS (OAPS), physicians are frequently faced with patients who do not meet these criteria. These cases are globally defined as non-criteria OAPS (NC-OAPS). In the literature, there are variations in the features included in this definition [4,5], and its management is controversial. The European Registry on Obstetric Antiphospholipid Syndrome (EUROAPS) cohort study, which compares 1000 women with diagnosis of OAPS and 640 with NC-OAPS, found clinical and laboratory disparities between patients of both groups, although fetal–maternal outcomes were similar when treatment was administered [5].

Adverse pregnancy outcomes (APO), such as sterility and recurrent implantation failure (RIF), are being investigated as related to aPLs. The prevalence of positive aPL in infertile women was reported in 0–7.6%, according to the aPL subtype [6], and in <5% for women with RIF [7]. Even though there is a low prevalence of aPLs in these settings, a recent review found that the relative risk for the presence of any type of aPL was 3.06 in women with RIF compared to women having at least one successful in vitro fertilization (IVF) [8]. By contrast, a previous metanalysis of prospective studies found no significant correlations between aPLs and IVF outcomes [9].

The types of aPLs implicated in APO are also a matter of debate. The PREGNANTS study is a multicenter study designed to assess the risk of obstetric complications in women with primary APS according to the aPL profile. The authors found that anti-β2glycoprotein I antibodies (aβ2GPI) were associated with the lowest live birth rate and highest incidence of preeclampsia (PE), fetal growth restriction (FGR), and stillbirth, compared with the presence of anticardiolipin (aCL) antibodies or lupus anticoagulant (LA) alone [10]. However, a more recent systematic review focusing on the association of aβ2GPI IgG and obstetric events found no association of this antibody with the risk of fetal loss, placental abruption, FGR, or composite obstetric complications, and the association between aβ2GPI IgG and PE/eclampsia was inconsistent [11]. Focusing on concrete APO, a metanalysis analyzing the association of the aPLs and late fetal loss found a statistically significant association between LA and late fetal loss (OR: 5.02, 95% confidence interval [CI] 2.14–7.89), but not for aCL (OR 3.47, 95% CI 0.68–6.26) or aβ2GPI (OR 3.13, 95% CI 0.75–5.50) [12]. A metanalysis analyzing the association of FGR and aPLs found a statistically significant association with aCL in cohort studies but not in case-control studies (OR: 2.35, 95% CI 1.59–3.11, and OR: 1.68, 95% CI 0.06–3.42, respectively); similar findings were demonstrated for aβ2GPI (OR 1.31, 95% CI 1.12–1.49) [13]. No association was found between FGR and LA (OR 0.82, 95% CI 0.54–1.10) [13].

Several studies have also suggested that low aPL titers have a similar clinical relevance in obstetric complications compared to medium or high titers [14]. For patients with clinical criteria for OAPS, but seronegative for conventional aPLs, an effort has arisen in the last two decades to discover new aPLs with clinical implications. Non-conventional aPLs were reported in 68% of these patients [15]. Anti-phosphatidylserine/prothrombin (aPS/PT) IgG/IgM antibodies are amongst the best studied, and many groups have showed their association with APO [15,16,17].

In women with OAPS, standard treatment consists of low dose aspirin (LDA) and low molecular weight heparin (LMWH) during pregnancy, which results in a rate of successful pregnancies up to 80–85% [18]. Other treatments, such as steroids, hydroxychloroquine (HCQ), or biologics (particularly, anti-TNF agents) were used, especially in refractory cases, but the data about their effectiveness is inconclusive and/or limited to case reports or case series [19,20,21,22]. Whether women with NC-OAPS require treatment is controversial. Both EUROAPS and EUREKA studies suggested that patients with NC-OAPS benefited from treatment [5,23]. Some studies found that patients with aPS/PT also have a reduction of APO if they receive treatment during their pregnancy [15,16,17]. Furthermore, recent retrospective studies found that treatment with LDA plus LMWH +/− HCQ improved IVF outcomes in patients with aPLs, increasing the clinical pregnancy, implantation, and take-home baby rates [24,25]. Given the difficulty of performing randomized controlled trials in this field, there are no appropriately designed studies that could better evaluate treatment effectiveness. Alternatively, potential valuable information may be achieved from real-world cohorts from dedicated centers.

The main objective of this study is to characterize the clinical and laboratory features, management, and pregnancy outcomes of women with APO who do not meet the Sydney clinical and/or laboratory APS criteria (NC-OAPS) in a monocentric real-world cohort. The secondary objective is to compare the cohort with a contemporaneous group of women with OAPS.

## 2. Materials and Methods

### 2.1. Patients

**Figure 1:**
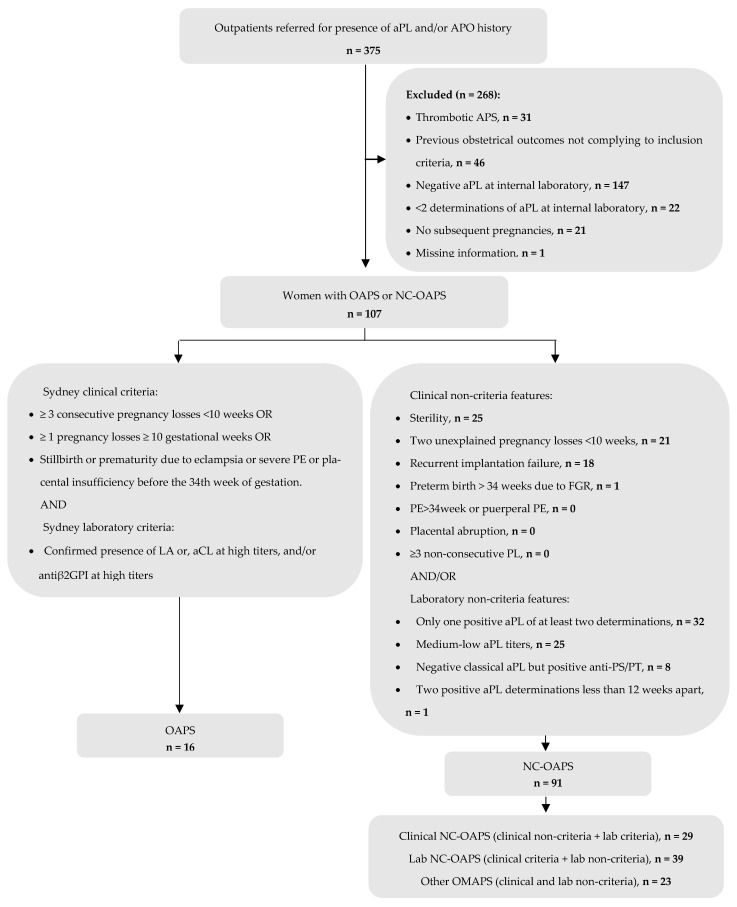
Study flowchart. Selected patients with OAPS and NC-OAPS from an initial cohort of women with aPLs and/or APO history. Abbreviations: aβ2GPI: anti-β2 glycoprotein-1 antibodies; aCL: anticardiolipin antibodies; aPL: antiphospholipid antibodies; APO: adverse pregnancy outcome; aPS/PT: anti-phosphatidylserine/prothrombin complex antibodies; FGR: fetal growth restriction; LA: lupus anticoagulant; NC-OAPS: non-criteria obstetric antiphospholipid syndrome; OAPS: obstetric antiphospholipid syndrome; OMAPS: obstetric morbidity related to APS; PE: preeclampsia.

This is a retrospective study of women referred to a high-risk obstetric unit of a tertiary hospital between 2010 and 2022 with APO (not attributed to anatomical, genetic, or infectious cause) and aPL positivity. The inclusion and exclusion criteria, as well as the number of patients in each corresponding group, are detailed on the flowchart of Figure 1. The OAPS group is composed with patients with obstetrical APS fulfilling the Sydney classification criteria [2]. The NC-OAPS group includes: patients with confirmed positive criteria aPL, but not meeting clinical Sydney criteria for OAPS (clinical NC-OAPS); or with clinical Sydney criteria and non-criteria aPL titers (lab NC-OAPS); or with non-criteria obstetric morbidity associated with non-criteria aPL titers or subtypes (obstetric morbidity related to APS [OMAPS] subgroup).

This study was approved by the Institutional Review Board at the Hospital Clinic, Barcelona (protocol code HCB/2018/1221, approved on 13 December 2018), with a waiver of informed consent given the retrospective nature of this study. This study conformed to the principles of the Declaration of Helsinki.

### 2.2. Recorded Information and Definitions

Medical records were reviewed retrospectively, and the following information was obtained:

- Demographic characteristics and previous medical history.
- aPLs: LA, determined according to the guidelines of the International Society of Thrombosis and Haemostasis [26], and IgG and IgM isotypes of aCL and aβ2GPI determined by enzyme-linked immunosorbent assay (ELISA) or by chemiluminescent immunoassay (CLIA). Samples were considered positive when ≥20 U/mL GPL/MPL if ELISA was used, or ≥20 chemiluminescent units (CU) for CLIA (equivalent to >99th percentile, according to the manufacturer). The defined ‘criteria titers’ were as follows: IgG/IgM aCL > 40 GPL/MPL by ELISA, IgG aCL > 95 CU or IgM aCL > 31 CU by CLIA (equivalent to antibody titers of 40 GPL/MPL by ELISA, according to Lakos et al. [27]), and aβ2GPI antibody results >40 GPL/MPL or ≥20 CU. Results above the cut-off value but below these titers were categorized as ‘low-medium, non-criteria titers’. aPS/PT IgG and IgM antibodies were determined by ELISA and results at the 99th percentile (≥30 U/mL) were considered positive, according to the manufacturer’s instructions.
- Subsequent pregnancies, which were those conceived after medical evaluation at our unit. For each subsequent pregnancy, the following data were collected: maternal age at delivery, treatment, and obstetric outcomes. The following relevant APO were defined and recorded:
  - early pregnancy loss (PL): spontaneous PL before week 10 of gestation
  - late PL: spontaneous PL of a morphologically normal fetus at or beyond week 10 of gestation
  - PE or eclampsia during gestation or puerperium
  - placental abruption
  - FGR: estimated fetal weight below the 3rd percentile for a given gestational age, or below the 10th percentile associated with Doppler abnormalities
  - premature delivery (before 37 weeks of gestation) because of placental vasculopathy
  - low birthweight (under 2500 g)
  - neonatal death before hospital discharge due to complications of prematurity and/or placental insufficiency.
- Additional outcomes, such as elective medical terminations, maternal thrombosis, hemorrhage or death, as well as embryo/fetal chromosomal analysis were also collected.

### 2.3. Statistical Analysis

Qualitative variables are expressed as absolute values or percentages. Their associations were evaluated using chi-square or Fisher’s exact tests, according to normal distribution. Continuous variables were expressed as a mean with standard deviation (SD), and t-tests were performed for their comparison. *p* values under 0.05 were considered significant. Statistical analyses were performed using SPSS version 20 (IBM SPSS Statistics 20).

## 3. Results

Among a total of 375 patients referred to the high-risk obstetric unit because of aPL positivity and/or previous APO, 107 women with 143 pregnancies (four twin) were included in the analysis (Figure 1). A total of 91 patients (85.0%) were classified as having NC-OAPS, while 16 (15.0%) had OAPS.

### 3.1. Characteristics of Patients with NC-OAPS

Twenty-four women (26.4%) had an additional autoimmune disease, most frequently autoimmune thyroiditis (9.9%), with controlled thyroid function. Six patients (6.6%) had an inherited thrombophilia: heterozygous factor V Leiden (*n* = 4), and heterozygous prothrombin gene mutation (*n* = 2). Unexplained sterility was the most frequent previous pregnancy morbidity reported (36.3%), followed by two or ≥ three early PL (24.2% each). Concerning aPL profile, aCL was the most and LA the least common positive aPL (Table 1), and around 50% of patients had single aPL positivity.

### 3.2. Subsequent Pregnancies in Patients with NC-OAPS: Treatment and Outcomes

There were 119 subsequent pregnancies in the follow-up of patients with NC-OAPS. The combination of LDA and LMWH was the most common treatment regimen used (87.4%). Treatment was maintained until the end of pregnancy except for six patients, in which LMWH was stopped after the first trimester. Corticosteroids and HCQ were used in 14.3% and 19.3% of pregnancies, respectively, only in combination with antithrombotic/anticoagulant treatment.

Most pregnancies ended successfully with the delivery of live newborns (82.4%). Early PL occurred in 18 patients (15.1%), of which 9 (50%) had confirmed aneuploidy on chorionic villus biopsy and 3 (16.7%) had confirmed euploidy; for 6 patients (33.3%) a chromosomal analysis was not performed. Late PL occurred twice in the same patient, at 12 and 13 gestational weeks, both with euploid chorionic villus biopsy results. PE occurred in 5.2% of women with NC-OAPS, all of them were non-severe and after 34 weeks of gestation.

Regarding fetal and neonatal outcomes, low birthweight and FGR were the most common complications observed (11.5% and 9.0%, respectively). Preterm births occurred because of severe HELLP (hemolysis, elevated liver enzymes and low platelets) syndrome in one pregnancy and FGR in two.

### 3.3. Comparison Between NC-OAPS and OAPS Groups

**Figure 2:**
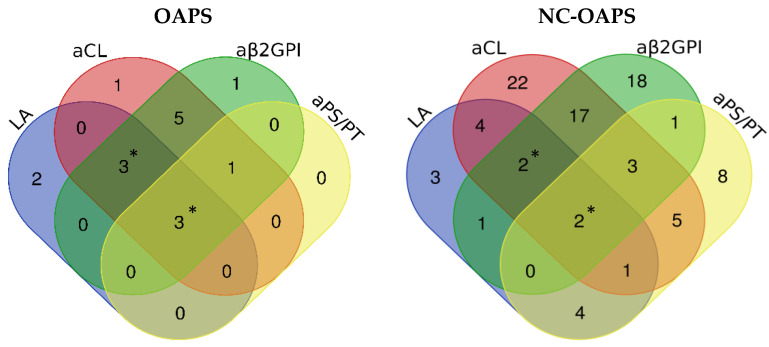
Composition and distribution of aPL subtypes in OAPS and NC-OAPS groups. Abbreviations: aβ2GPI: anti-β2 glycoprotein-1 antibodies; aCL: anticardiolipin antibodies; aPS/PT: anti-phosphatidylserine/prothrombin complex antibodies; LA: lupus anticoagulant; NC-OAPS: non-criteria obstetric antiphospholipid syndrome; OAPS: obstetric antiphospholipid syndrome. * *p* < 0.05 for the comparison between percentages within OAPS and NC-OAPS.

No statistically significant differences were recorded between patients with NC-OAPS and women with OAPS in terms of ethnicity, cardiovascular risk factors, and associated autoimmune diseases (Table 2). Considering all the previous pregnancy morbidity each patient experienced, late PL was the most frequent previous APO in the OAPS group (62.5%), contrasting with unexplained sterility in the NC-OAPS group (36.3%). History of a late PL was significantly more common in women with OAPS, whereas RIF was significantly more frequent in NC-OAPS. Positivity of aβ2GPI and LA was more frequent in the OAPS group, while the other aPL subtypes had comparable frequencies between groups. Triple or quadruple aPL positivity were more frequent in the OAPS group (Table 2 and Figure 2).

There were 24 subsequent pregnancies in patients with OAPS. Assisted reproduction techniques were used in 6 (25%) of patients with OAPS and 55 (46.6%) of those with NC-OAPS (*p* = 0.070). Treatment with the combination of LDA and LMWH was used with similar frequencies in OAPS and NC-OAPS (83.3% versus 87.4%, *p* > 0.05) (Table 3). Delivery of live newborns occurred in comparable percentages in pregnancies in OAPS (75.0%) and NC-OAPS (82.4%) groups, *p* > 0.05 (Table 3). Considering all subsequent pregnancies per woman, after evaluation at our unit, 100% of women with OAPS achieved the birth of at least one live newborn, a percentage similar to those with NC-OAPS (93.4%, *p* > 0.05).

The occurrence of at least one APO per pregnancy was comparable between groups (37.5% in OAPS vs. 31.6% in NC-OAPS, *p* > 0.05). No significant differences were found between groups in terms of early or late PL, PE, preterm birth, or low birthweight neonates (Table 3). The indications for caesarean section were predominantly obstetric in nature. In five cases, caesarean section was performed because of placental insufficiency: placental abruption (*n* = 1, in a patient with NC-OAPS), PE (*n* = 2, both in women with OAPS), FGR (*n* = 2, in the NC-OAPS group). There were no maternal thrombosis or maternal deaths in any group.

## 4. Discussion

This study reflects a real-world scenario of a specialized obstetric unit in a third level hospital and shows that patients with NC-OAPS correspond to more than 80% of patients referred. The results of this study show an overall good pregnancy outcome for women with NC-OAPS when treatment is offered, comparable to patients with treated OAPS.

Our study found that a previous history of late PL was significantly more common in women with OAPS, whereas RIF was more common in NC-OAPS. These observations are in line with the results of the EUROAPS study, in which patients with OAPS had a higher number of early PL, late PL, stillbirth, early placental vasculopathy, and prematurity than those with NC-OAPS, whereas women with NC-OAPS had higher rates of RIF and late placental events [5]. Placental vasculopathy was rare in our cohort, which could contribute to the absence of prevalence differences between NC-OAPS and OAPS groups. Furthermore, we found a high percentage of sterility in our cohort, a variable that was not included in the EUROAPS study. This may indicate that, in routine clinical practice, patients with sterility are increasingly investigated for the presence of aPLs and, if positive, referred for management. Nevertheless, there is a gap in the literature respecting this issue and further research is required.

Patients with NC-OAPS are more likely to have single aPL positivity, in contrast with a higher number of positive aPLs in OAPS patients. Patients in the EUROAPS study were similar to those of our cohort. In that study, patients with double or triple aPL positivity and patients with LA positivity alone more frequently had OAPS. Patients with aCL and aβ2GPI more frequently had NC-OAPS [5]. In our study, LA was also significantly more frequent in OAPS but, contrary to the EUROAPS study, aβ2GPI was also more common in OAPS, in part possibly because of the low thresholds used as criteria titers of this antibody when determined by CLIA in our study.

The optimal treatment in NC-OAPS patients is not defined. In our series, more than 80% of patients with NC-OAPS were treated with a combination of LDA and LMWH, a frequency similar to that of patients with OAPS. These results are aligned with those of a systematic review, aiming to compare treatment regimens in NC-OAPS versus OAPS [28]. The biologic mechanisms involved in the pathogenesis of aPL-associated obstetric morbidity may include not only thrombosis but trophoblast dysfunction, as well as decidual inflammation, complement activation, and implication of neutrophil extracellular traps [16]. For these reasons, apart from anti-thrombotic drugs and heparin, other treatments are commonly used, particularly in refractory OAPS [29], but their efficacy is yet to be clarified. We reported the adjuvant use of HCQ and corticosteroids in 8–20% of subjects, with the NC-OAPS group tending to receive these additional therapies more commonly. Other studies have reported a higher use of HCQ and steroids in NC-OAPS [30].

In subsequent pregnancies, our study found a similar prevalence of live births and APO between women with NC-OAPS and OAPS. Our results coincide partially with that of EUROAPS, as both groups had similar treatment percentages and live birth rates. However, the EUROAPS study [5] reported a globally higher APO incidence (73.4% in NC-OAPS and 65.1% in OAPS) compared to our study (31.6% in NC-OAPS and 37.5% in OAPS). It is noteworthy that, in the EUROAPS study, the specific events included in the composite APO definition are not clearly delineated. Furthermore, as a multicenter study, variations in event definitions and frequencies across centers may have contributed to these differences. A metanalysis reported that five studies described an improvement of live births in both NC-OAPS and OAPS with treatment [28]. Several other studies of women with NC-OAPS, defined in a similar manner as in our study, suggested that they have similar pregnancy outcomes with standard treatment for OAPS [5,16,23,31,32,33]. On the whole, an interpretation of the results should be made with caution, as NC-OAPS definition varies among studies and most study designs do not control the exposure to treatment.

An important focus of the scientific community in this area has been to identify prognostic factors for new obstetric complications and hopefully tailoring the management [34]. Research groups developed scores focusing on aPLs to stratify the risk of patients carrying these antibodies, such as the Global Antiphospholipid Syndrome Score (GAPSS) score [35], its variant aGAPSS score [36], and the EUREKA algorithm [23]. Few studies evaluated the prediction value of GAPSS/aGAPSS for pregnancy morbidity, and the results are contradictory [37,38,39]. The authors of the EUREKA algorithm found that the probability of developing APO in women with aPLs was 64% with low titers and 68% with high titers, with a higher risk in those with LA and/or IgG antiβ2GPI positivity [23]. All women with low-titer aPLs benefited from treatment with LDA + LMWH ± HCQ, and its effectiveness was even greater for low-titer than for criteria aPL [23]. Nowadays, there is still no consensus about how to stratify the obstetric risk and tailor treatment, particularly in non-criteria subjects and after new obstetric events. New technologies are being used to explore genetic polymorphisms that eventually, in the future, can be used as biomarkers for clinical phenotypes and prognostic tools [40].

Recurrent early PL is the obstetric complication for which a diagnosis of APS is most often considered, and it was the most frequent subsequent APO observed in our study. However, the role of aPLs in early PL is not always clearly defined, given the lack of discrimination in the literature between biochemical and clinical losses, and incomplete evaluations for other causes of PL. Reflecting this last limitation, we reported that, within the 23 early PL occurring in our entire cohort, only 13 had unknown cause, as the other 10 (9 in NC-OAPS and 1 in OAPS) were associated with a known embryo aneuploidy. Not performing an embryonic/fetal chromosomal analysis could overestimate the magnitude of aPL effect in these events and eventually indicate a false therapeutic failure.

Our study has limitations. It is retrospective, with inherent constraints in data collection and analysis, such as the variation of laboratory methods used to test for aPLs along time, and unstandardized treatment regimens. One of the most important is that the aPL detection methods varied between and within patients. CLIA has been the only method used at our laboratory since 2015. We acknowledge that this supposes a research limitation, as CLIA is not the standard method used to classify a patient with APS, and the correlation of values between the moderate–high thresholds of ELISA and CLIA is variable for different aPL subtypes and still requires validation. As there is ambiguity about the cut-off values for low-titers and criteria titers of aPLs detected by CLIA, we tried to overcome this limitation by using the equivalent aCL criteria titers according to Lakos et al. [27]. Nevertheless, for the aβ2GPI subtype, criteria levels were the in-house cut-off values. According to Vandevelde et al. [41], the use of these CLIA units would overestimate the number of patients with criteria aPL titers and eventually amplify the OAPS group, which is in fact the smaller group of our study. Another limitation of our study is the large difference in the size of the two cohorts analyzed, which may affect the validity of the comparison between them. The smaller size of the OAPS cohort at the same lapse of time may be attributed to several factors. On the one hand, patients are increasingly referred for a medical visit for APO not fulfilling the clinical APS criteria (for example, after two PL), and treatment is equally initiated, which potentially avoids another APO in the next pregnancy and the eventual classification of patients with OAPS. On the other hand, we strictly used the Sidney criteria for attribution of the OAPS diagnosis. Since these are classification criteria, it is possible that, in clinical practice, many patients are diagnosed with and managed as OAPS without fulfilling them. Given the uncertainties of the impact of aPLs in non-criteria pregnancy morbidity and of different aPL titers and subtypes, the classification criteria are increasingly restrictive regarding the clinical features included (according to ACR/EULAR 2023 criteria, only eight patients in this study would have been classified as OAPS), while physicians in daily clinical practice seem to take an opposite direction, assuming (and treating) the relevance of low or transitory aPL titers in otherwise unexplained pregnancy morbidity. Classification criteria are specifically designed to ensure high specificity, thereby identifying homogeneous populations for research purposes. Consequently, they do not encompass the full clinical spectrum of a disease but instead focus on patients exhibiting its hallmark features. Meeting the classification criteria does not confirm an APS diagnosis, as the observed adverse event may stem from factors unrelated to aPLs. Similarly, failure to meet the criteria does not exclude APS but may reflect an uncertain or probable diagnosis. Identifying such cases and exploring optimal treatment strategies for these patients remains critical [42]. Within this framework, our study contributes further evidence supporting the effectiveness of treatment in this subgroup of women.

We consider that our study has several strengths. We included a cohort of women that represents the whole spectrum of patients with a clinical suspicion of aPL-associated pregnancy morbidity. Patients with thrombotic APS were excluded, providing a more homogenous group of patients only with unfavorable obstetric history. Finally, some of the PL observed were submitted for chromosomal analysis, revealing a not negligible number of aneuploidies. When feasible, a cytogenetic analysis of products of conception should be performed in all pregnancy losses to avoid erroneous tagging of aPL-related morbidity.

## 5. Conclusions

Patients with non-criteria obstetric antiphospholipid syndrome represent an important and frequent, although incompletely known group in routine clinical practice. According to our results, patients with non-criteria obstetric antiphospholipid syndrome may be treated similarly to those with obstetric antiphospholipid syndrome. The outcome of subsequent pregnancies in treated patients is good and similar to treated patients with obstetric antiphospholipid syndrome.

## Figures and Tables

**Table 1:** Demographic features, previous APO and aPL profile of patients with NC-OAPS.

Demographic and Clinical Characteristics	NC-OAPS Patients (n = 91)
Ethnicity, n (%):	
Caucasian	81 (89.0)
Hispanic/South American	5 (5.5)
Maghrebi	1 (1.1)
Asian	3 (3.3)
Black	1 (1.1)
BMI, mean (S.D.) ^a^	24.73 (4.61)
Smoking habits, n (%) ^a^	18 (20.0)
Arterial hypertension, n (%)	1 (1.1)
Diabetes mellitus, n (%)	1 (1.1)
Dyslipidemia, n (%)	1 (1.1)
Presence of other autoimmune diseases, n (%):	24 (26.4)
Autoimmune thyroiditis	9 (9.8)
Systemic lupus erythematosus	3 (3.3)
Rheumatoid arthritis	2 (2.2)
Psoriasis	2 (2.2)
Cutaneous lupus	1 (1.1)
Systemic sclerosis	1 (1.1)
Sjögren’s syndrome	1 (1.1)
Undifferentiated connective tissue disease	1 (1.1)
Seronegative spondyloarthropathy	1 (1.1)
Immune thrombocytopenic purpura	1 (1.1)
IgA vasculitis	1 (1.1)
Multiple sclerosis	1 (1.1)
Inherited thrombophilia, n (%)	6 (6.6)
**Previous APO**	
2 early pregnancy losses, n (%)	22 (24.2)
≥3 early pregnancy losses, n (%)	22 (24.2)
≥1 late pregnancy loss, n (%)	16 (17.6)
Neonatal death (because of FGR/PE/prematurity), n (%)	2 (2.2)
Preterm birth <34 weeks because of FGR, n (%)	3 (3.3)
Preterm birth >34 weeks because of FGR, n (%)	2 (2.2)
PE/eclampsia <34 weeks, n (%)	2 (2.2)
PE/eclampsia >34 weeks, n (%)	1 (1.1)
Placental abruption, n (%)	2 (2.2)
Recurrent implantation failure, n (%)	20 (22.0)
Unexplained sterility, n (%)	30 (36.3)
**Antiphospholipid antibodies subtypes**	
Lupus anticoagulant, n (%)	17 (18.7)
aCL IgM and/or IgG autoantibodies, n (%)	56 (61.5)
aβ2GPI IgM and/or IgG autoantibodies, n (%)	44 (48.4)
aPS/PT IgM and/or IgG autoantibodies, n (%) ^b^	24 (51.1)

Abbreviations: aβ2GPI: anti-β2 glycoprotein-1 antibodies; aCL: anticardiolipin antibodies; APO: adverse pregnancy outcomes; aPS/PT: anti-phosphatidylserine/prothrombin complex antibodies; BMI: body mass index; FGR: fetal growth restriction; NC-OAPS: non-criteria obstetric antiphospholipid syndrome; PE: preeclampsia; S.D.: standard deviation; w: weeks. ^a^ BMI data were available in 85 patients and smoking habits in 90 patients. ^b^ aPS/PT determined in 47 patients (missing in 44).

**Table 2:** Comparison of demographic, obstetric history and aPL profiles in women with NC-OAPS and women with OAPS.

	NC-OAPS (91)	OAPS (n = 16)	*p* Value
**Demographic and clinical characteristics**			
Ethnicity, n (%)			
Caucasian	81 (89.0%)	12 (75.0%)	0.201
Hispanic/South American	5 (5.5%)	2 (12.5%)	
Maghrebi	1 (1.1%)	1 (6.2%)	
Asian	3 (3.3%)	1 (6.2%)	
Black	1 (1.1%)	0 (0%)	
BMI, mean (S.D.) ^a^	24.73 (4.61)	25.03 (6.10)	0.830
Smoker, n (%) ^a^	18 (20.0)	1 (6.7)	0.296
Arterial hypertension, n (%)	1 (1.1)	2 (12.5)	0.058
Diabetes mellitus, n (%)	1 (1.1)	0 (0)	1.000
Dyslipidemia, n (%)	1 (1.1)	0 (0)	1.000
Presence of other autoimmune diseases, n (%)	24 (26.4)	4 (25.0)	1.000
Inherited thrombophilia, n (%)	6 (6.6)	0 (0)	0.588
Age at first subsequent pregnancy, years, mean (S.D.)	36.86 (3.94)	36.81 (6.41)	0.979
Subsequent pregnancies per patient, mean (S.D.)	1.31 (0.59)	1.5 (0.82)	0.261
**Previous obstetric history**			
Live births before study, n (%)	18 (19.8)	7 (43.8)	0.053
2 early pregnancy losses, n (%)	22 (24.2)	1 (6.2)	0.184
≥3 early pregnancy losses, n (%)	22 (24.2)	3 (18.8)	0.758
≥1 late pregnancy loss, n (%)	16 (17.6)	10 (62.5)	0.000
Neonatal death (because of FGR/PE/prematurity), n (%)	2 (2.2)	2 (12.5)	0.105
Previous preterm birth <34 weeks because of FGR, n (%)	3 (3.3)	1 (6.2)	0.482
Previous preterm birth >34 weeks because of FGR, n (%)	2 (2.2)	0 (0)	1.000
PE/eclampsia <34 weeks, n (%)	2 (2.2)	2 (12.5)	0.105
PE/eclampsia >34 weeks, n (%)	1 (1.1)	1 (6.2)	0.278
Placental abruption, n (%)	2 (2.2)	0 (0)	1.000
Recurrent implantation failure, n (%)	20 (22.0)	0 (0)	0.038
Unexplained sterility, n (%)	30 (36.3)	2 (12.5)	0.062
**aPL profile**			
Subtypes of aPL			
Lupus anticoagulant, n (%)	17 (18.7)	8 (50.0)	0.011
aCL autoantibodies, n (%)	56 (61.5)	13 (81.2)	0.129
IgM	14 (25.0)	3 (23.1)	1.000
IgG	37 (66.1)	8 (61.5)	0.756
IgM + IgG	5 (8.9)	2 (15.4)	0.609
aβ2GPI autoantibodies, n (%) ^b^	44 (48.4)	13 (86.7)	0.006
IgM	17 (38.6)	3 (23.1)	0.346
IgG	24 (54.5)	7 (53.8)	0.965
IgM + IgG	3 (6.8)	3 (23.1)	0.125
aPS/PT autoantibodies, n (%) ^c^	24 (51.1)	4 (50.0)	1.000
IgM	20 (85.7)	3 (75.0)	1.000
IgG	1 (4.2)	0 (0)	1.000
IgM + IgG	3 (12.5)	1 (25.0)	0.481
Number of positive aPL tests			
In patients with aPS/PT determination (n = 55):			
Single positive, n (%)	27 (57.4)	2 (25.0)	0.131
Double positive, n (%)	14 (29.8)	2 (25.0)	1.000
Triple positive, n (%)	4 (8.5)	1 (12.5)	0.559
Four positive, n (%)	2 (4.3)	3 (37.5)	0.018
In patients without aPS/PT determination (n = 52):			
Single positive, n (%)	24 (54.5)	2 (25.0)	0.248
Double positive, n (%)	18 (40.9)	3 (37.5)	1.000
Triple positive, n (%)	2 (4.5)	3 (37.5)	0.022
aGAPSS ^b^, n (%)			
≤5	61 (67.0)	4 (26.7)	0.003
>5	30 (33.3)	11 (73.3)	

Abbreviations: BMI: body mass index; aβ2GPI: anti-β2 glycoprotein-1 antibodies; aCL: anticardiolipin antibodies; aGAPSS: adjusted Global APS score; aPL: antiphospholipid antibodies; aPS/PT: anti-phosphatidylserine/prothrombin complex antibodies; FGR: fetal growth restriction; NC-OAPS: non-criteria obstetric antiphospholipid syndrome; OAPS: obstetric antiphospholipid syndrome; PE: preeclampsia; S.D.: standard deviation. ^a^ BMI was missing in 6 patients with NC-OAPS and 2 with OAPS, and smoking habits were missing in one patient in each group. ^b^ aβ2GPI and aGAPSS was determined in 106 patients (missing data in one with OAPS). ^c^ aPS/PT was determined in 55 patients (missing in 8 patients with OAPS and 44 with NC-OAPS).

**Table 3:** Obstetric, maternal, and fetal/neonatal outcomes among subsequent pregnancies in women with NC-OAPS and women with OAPS.

	NC-OAPS n = 119	OAPS n = 24	*p* Value
**Treatment regimen**			
No treatment, n (%)	1 (0.8)	0 (0)	1.000
LDA alone, n (%)	12 (10.1)	3 (12.5)	0.718
LMWH alone, n (%)	2 (1.7)	1 (4.2)	0.426
LDA plus LMWH, n (%)	104 (87.4)	20 (83.3)	0.527
**Additional treatments**			
Hydroxychloroquine, n (%)	23 (19.3)	2 (8.3)	0.250
Corticosteroids, n (%)	17 (14.3)	2 (8.3)	0.741
ART, n (%)	55 (46.6%)	6 (25.0)	0.070
**Treatment doses**			
Aspirin 100 mg, n (%)	103 (86.6)	15 (62.5)	0.015
Aspirin 150 mg, n (%)	13 (10.1)	8 (33.3)	0.009
Prophylactic doses LMWH, n (%)	92 (77.3)	15 (62.5)	0.127
Intermediate doses LMWH, n (%)	10 (8.4)	6 (25.0)	0.030
Therapeutic doses LMWH, n (%)	4 (3.4)	0 (0)	1.000
Corticosteroids ≥ 10 mg/day, n (%)	13 (10.9)	1 (4.2)	0.465
**Final pregnancy outcome**			
Live births, n (%)	98 (82.4)	18 (75.0)	0.400
Elective medical termination, n (%)	1 (0.8)	1 (4.2)	0.308
Pregnancy loss < 10 w, n (%)	18 (15.1)	5 (20.8)	0.543
Pregnancy loss 10–24 w, n (%)	2 (1.7)	0 (0)	1.000
Pregnancy loss > 24 w, n (%)	0 (0)	0 (0)	NA
**Obstetric and maternal outcomes**			
Deliveries, n	98	18	
Caesarean section, n (%) ^a^	51 (53.1)	5 (29.4)	0.071
Preeclampsia, n (%) ^b^	5 (5.2)	3 (16.7)	0.111
HELLP, n (%) ^b^	1 (1.0)	0 (0)	1.000
Placental abruption, n (%) ^b^	1 (1.0)	0 (0)	1.000
Maternal death, n (%)	0 (0)	0 (0)	NA
Maternal thrombosis, n (%)	0 (0)	0 (0)	NA
Postpartum hemorrhage	4 (4.6)	0 (0)	1.000
**Fetal and neonatal outcomes**			
Neonates, n	n = 101 *	n = 18	
Gestational Age (weeks), mean (SD)	38.791 (2.00)	38.6 (1.88)	0.653
FGR, n (%) ^c^	9 (9.0)	0 (0)	0.351
Preterm birth related to PE/HELLP/FGR, n (%) ^c^	3 (3.0)	1 (5.6)	0.489
<28 weeks	1	0	
32–33.6 weeks	0	1	
34–<37 weeks	2	0	
Birthweight (gr), mean (SD)	3050 (550)	3146 (506)	0.491
Low birthweight, n (%) ^c^	11 (11.5)	2 (11.1)	1.000
Neonatal death, n (%)	1 (1.0)	0 (0)	1.000

Abbreviations: FGR: fetal growth restriction; HELLP: Hemolysis, Elevated Liver enzymes and Low Platelets; LDA: low-dose aspirin; LMWH: low molecular weight heparin; NA: not applied; NC-OAPS: non-criteria obstetric antiphospholipid syndrome; OAPS: obstetric antiphospholipid syndrome; PE: preeclampsia; SD: standard deviation; w: weeks. ^a^ Information for mode of delivery is missing in one woman in OAPS group and two women in the NC-OAPS group. ^b^ Data for preeclampsia, HELLP, and placental abruption are missing for two women in the NC-OAPS group. ^c^ Information for FGR and preterm birth is missing in one and for low birthweight is missing for five neonates in the NC-OAPS group. * Four twin pregnancies, one of which with loss of one fetus.

## Data Availability

The data underlying this article will be shared on reasonable request to the corresponding author.
